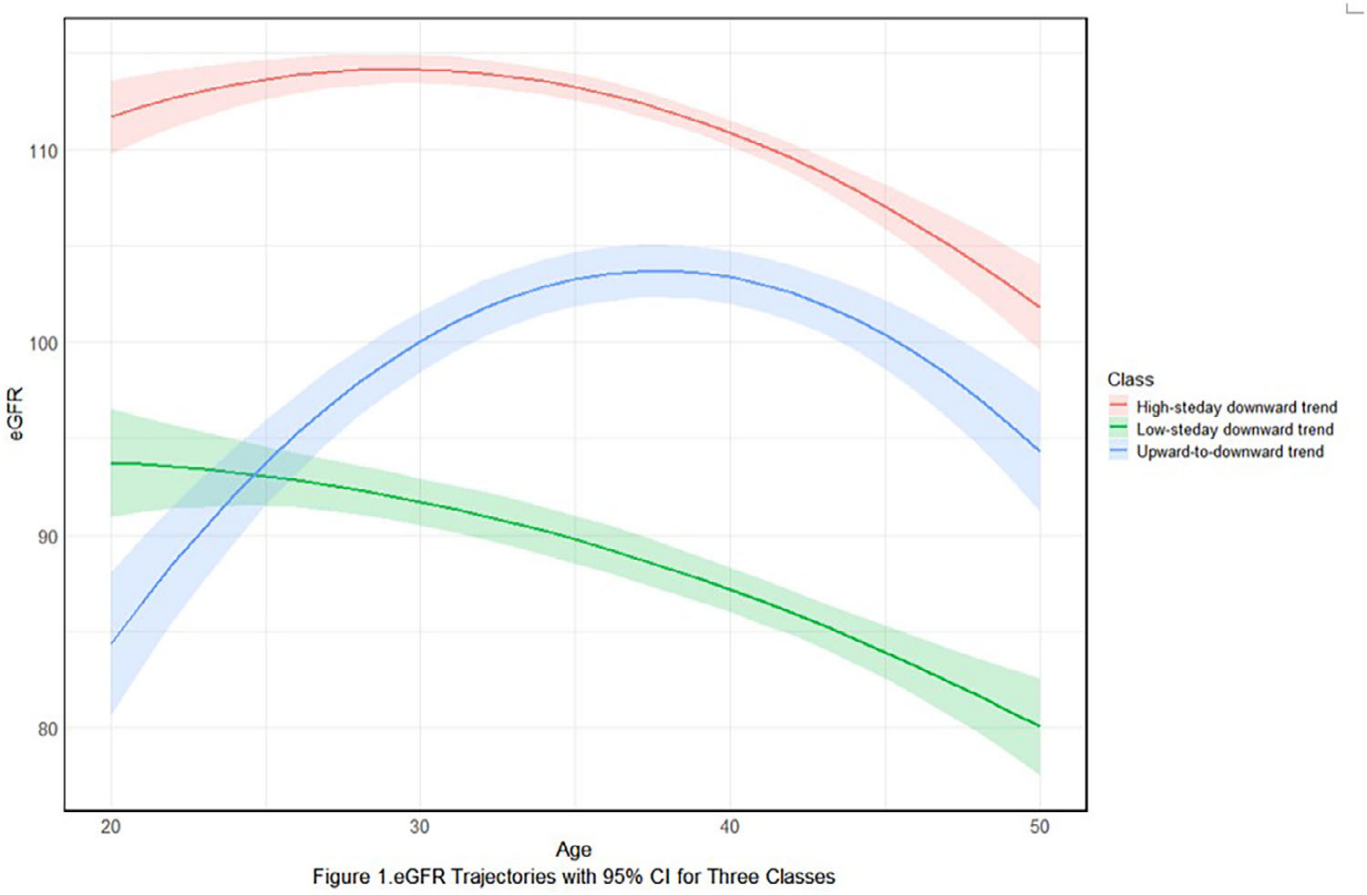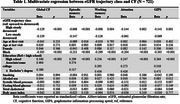# Association between eGFR Trajectories and Cognitive Function in Midlife: Longitudinal Findings from the Bogalusa Heart Study

**DOI:** 10.1002/alz70857_100091

**Published:** 2025-12-24

**Authors:** Eunsun Gill, Flor Alvarado, David J. Libon, Ileana De Anda‐Duran, Xuanyi Jin, Soo Jung Kang, Lydia A Bazzano, Camilo Fernandez‐Alonso, Emily Harville

**Affiliations:** ^1^ Celia Scott Weatherhead Tulane University School of Public Health and Tropical Medicine, New Orleans, LA, USA; ^2^ Tulane University School of Medicine, New Orleans, LA, USA; ^3^ Rowan University, Glassboro, NJ, USA; ^4^ Rowan‐Virtua School of Osteopathic Medicine, New Jersey Institute for Successful Aging, Stratford, NJ, USA

## Abstract

**Background:**

Estimated glomerular filtration rate (eGFR) (normally ranged at 90 mL/min/1.73m^2^) has a reported nonlinear association with cognitive function (CF). However, the relationship between eGFR trajectories over the life course and CF remains unclear. This study examines the association between eGFR trajectories and midlife CF using data from the Bogalusa Heart Study (BHS).

**Method:**

A total of 721 BHS participants (62.9% female, 30.6% Black, mean follow‐up: 17.2 ± 5.2 years) with at least three creatinine measurements in adulthood were included. eGFR was calculated using the 2021 CKD‐EPI Creatinine equation, and latent class growth analysis identified eGFR trajectory classes. CF was assessed with tests of verbal episodic memory, working memory, attention, graphomotor information processing speed (GIPS), and global CF. The global CF was computed by averaging all cognitive test scores. Multivariate regression examined the association between eGFR trajectory classes and CF, adjusting for sex, race, education, age at first and last visit, smoking, alcohol use, systolic blood pressure, glucose, total cholesterol, and body mass index, each summarized using the area under the curve.

**Result:**

Three eGFR trajectory classes were identified: “high‐steady downward trend” (46.7%), “upward‐to‐downward trend” (28.7%), and “low‐steady downward trend” (24.4%). Individuals with high and low eGFR trajectories had lower global CF than the upward‐to‐downward trend trajectory (β: –0.129 and β: –0.139; *p* <0.001). High‐steady downward trend trajectory was associated with lower episodic memory (β: –0.068), working memory (β: –0.098), attention (β: –0.144), and GIPS scores (β: –0.141). Low‐steady downward trend trajectory was associated with lower episodic memory (β: –0.147, *p* <0.001), working memory (β: –0.098, *p* = 0.004), attention (β: –0.144, *p* = 0.002), and GIPS (β: –0.141, *p* = 0.001).

**Conclusion:**

Our findings suggest that both high‐steady downward and low‐steady downward trend eGFR trajectories during adulthood are associated with worse global CF and cognition across all subdomains in midlife compared to the upward‐to‐downward trend trajectory. These results underscore the importance of maintaining stable renal function throughout adulthood to support cognitive health.